# The Potential Benefits of Quercetin for Brain Health: A Review of Anti-Inflammatory and Neuroprotective Mechanisms

**DOI:** 10.3390/ijms24076328

**Published:** 2023-03-28

**Authors:** Ming-Chang Chiang, Tsung-Yu Tsai, Chieh-Ju Wang

**Affiliations:** 1 Department of Life Science, College of Science and Engineering, Fu Jen Catholic University, New Taipei City 242062, Taiwan; 2 Department of Food Science, Fu Jen Catholic University, New Taipei City 242062, Taiwan

**Keywords:** quercetin, anti-inflammatory agents, AMPK, neuroprotection, NF-κB, NLRP3 inflammasome

## Abstract

Neuroinflammation is a critical factor in developing and progressing numerous brain diseases, including neurodegenerative diseases. Chronic or excessive neuroinflammation can lead to neurotoxicity, causing brain damage and contributing to the onset and progression of various brain diseases. Therefore, understanding neuroinflammation mechanisms and developing strategies to control them is crucial for treating brain diseases. Studies have shown that neuroinflammation plays a vital role in the progression of neurodegenerative diseases, such as Alzheimer’s (AD) and Parkinson’s (PD), and stroke. Additionally, the effects of PM_2.5_ pollution on the brain, including neuroinflammation and neurotoxicity, are well-documented. Quercetin is a flavonoid, a plant pigment in many fruits, vegetables, and grains. Quercetin has been studied for its potential health benefits, including its anti-inflammatory, antioxidant, and anti-cancer properties. Quercetin may also have a positive impact on immune function and allergy symptoms. In addition, quercetin has been shown to have anti-inflammatory and neuroprotective properties and can activate AMP-activated protein kinase (AMPK), a cellular energy sensor that modulates inflammation and oxidative stress. By reducing inflammation and protecting against neuroinflammatory toxicity, quercetin holds promise as a safe and effective adjunctive therapy for treating neurodegenerative diseases and other brain disorders. Understanding and controlling the mechanisms of NF-κB and NLRP3 inflammasome pathways are crucial for preventing and treating conditions, and quercetin may be a promising tool in this effort. This review article aims to discuss the role of neuroinflammation in the development and progression of various brain disorders, including neurodegenerative diseases and stroke, and the impact of PM_2.5_ pollution on the brain. The paper also highlights quercetin’s potential health benefits and anti-inflammatory and neuroprotective properties.

## 1. Neuroinflammation Toxicity and Neuroprotection

**Figure 1:**
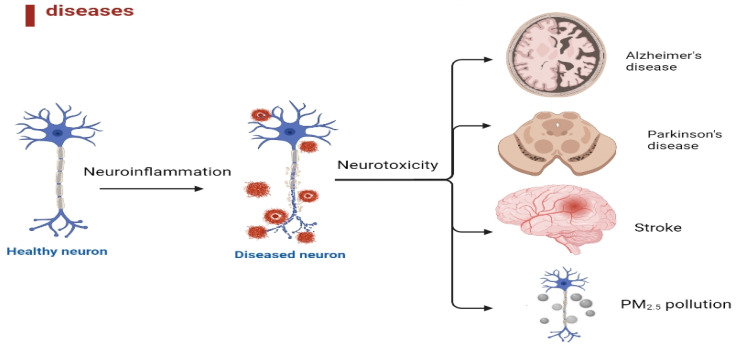
Neuroinflammation plays a crucial role in the pathological processes of AD, PD, stroke, and PM_2.5_ pollution in the brain.

Neuroinflammation is when the immune system reacts to injury or disease in the nervous system [1,2]. Neuroinflammation can occur as a response to the injury or disease, which can then contribute to further damage and degeneration in Alzheimer’s disease (AD), Parkinson’s disease (PD), stroke, and PM_2.5_ pollution (Figure 1).

In some cases, the initial lesion itself may not result from neuroinflammation results. Still, the resulting inflammatory response can exacerbate the injury and lead to further damage. Therefore, it is accurate to say that neuroinflammation is an essential mechanism in developing and progressing many brain lesions, but it is not the only mechanism. Inflammatory processes in the brain can cause damage to neurons, disrupt normal brain function, and lead to the progression of these diseases [3,4]. However, it is important to note that the relationship between neuroinflammation and neurodegenerative diseases is complex and not fully understood. Multiple factors can contribute to the development and progression of these conditions. Neuroprotection refers to strategies or interventions that can help to protect the nervous system from injury or disease, such as reducing inflammation, promoting the growth and survival of neurons, or removing toxic substances from the brain [5].

### 1.1. Neuroinflammation Plays a Crucial Role in the Pathological Processes in the Brain

Neuroinflammation can lead to a range of pathological processes, including cell death, changes in gene expression, and altered neurotransmitter function, among others [6,7]. These changes can contribute to developing and progressing neurological disorders and neurodegenerative diseases. When injury or disease occurs in the brain or spinal cord, both neurons and glial cells can become activated and release inflammatory mediators. This can lead to the recruitment of immune cells to the site of injury and the formation of a neuroinflammatory response. This neuroinflammatory response is a complex process that involves the activation and interaction of different types of cells and molecules, including microglia, astrocytes, and cytokines [8]. This can lead to the release of toxic molecules, the formation of reactive oxygen species, the activation of programmed cell death pathways, and the alteration of the blood-brain barrier (BBB) permeability. Therefore, understanding the role of neuroinflammation in the development of pathology (Figure 1) is important to identify potential targets for intervention and develop treatments that can help reduce inflammation, protect the neurons, and promote the repair of neural tissue.

Neuroinflammation is a complex process that involves the activation of immune cells and the release of inflammatory mediators in response to various stimuli in the brain [9]. It has been implicated in the pathogenesis of several neurodegenerative disorders such as AD, PD, stroke, and exposure to PM_2.5_ pollution. Neuroinflammation is a common thread linking several neurological disorders and environmental factors, and targeting this process may hold promise for developing new therapies for these conditions. The image was created in BioRender.

### 1.2. Inflammation Can Alter Cellular Functions in a Variety of Ways

During an inflammatory response, immune cells release cytokines and other signaling molecules that can affect the cells and tissues [10]. For example, inflammation can alter the permeability of blood vessels, leading to changes in blood flow and oxygenation, and it can also stimulate cells to produce harmful reactive oxygen species. Inflammation can also cause changes in gene expression, disrupt normal cellular processes, and contribute to the death of cells. These changes can significantly impact the function and survival of cells and tissues and contribute to the development of various diseases and disorders. Mitochondrial dysregulation refers to the disruption of normal mitochondrial function, which can lead to the production of reactive oxygen species and the loss of energy production. This can contribute to the development of neurotoxicity [11]. This can lead to the accumulation of toxic molecules that can damage cells and contribute to the development of neurotoxicity. Endoplasmic reticulum (ER) stress refers to the disruption of normal ER function, which can accumulate misfolded proteins and activate cell death pathways [12]. This can also contribute to the development of neurotoxicity. Cell signaling refers to the communication between cells necessary for normal cellular function. All these mechanisms can work together to promote inflammation and neurotoxicity [13], and it is important to understand the role of each mechanism in the development of neurological disorders to identify potential targets for intervention and to develop therapies that can help to reduce inflammation and protect the brain from injury and disease.

### 1.3. The Pathophysiology of Neuroinflammatory Toxicity in Neurodegenerative Diseases, Stroke, and the Role of PM_2.5_ Pollution Is a Complex and Multi-Factorial Process

AD is a progressive neurodegenerative disorder affecting millions worldwide. The pathogenesis of AD is complex and involves several molecular and cellular mechanisms. Beta-amyloid (Aβ) accumulation: Aβ is a peptide that accumulates in the brain of AD patients and is the major component of senile plaques [14]. The accumulation of Aβ is thought to be the primary cause of AD pathology. It leads to the formation of toxic oligomers and fibrils that disrupt neuronal function and cause neurodegeneration. In addition, in AD, tau undergoes abnormal hyperphosphorylation and aggregation, forming neurofibrillary tangles (NFTs) that disrupt neuronal function and cause cell death [14]. Neuroinflammation: Chronic inflammation is a hallmark of AD pathology. Activated microglia and astrocytes produce inflammatory cytokines and chemokines that contribute to neurodegeneration and exacerbate Aβ and tau pathology [15]. In summary, the pathogenesis of AD involves multiple molecular and cellular mechanisms, including Aβ accumulation, tau protein aggregation, neuroinflammation, oxidative stress, mitochondrial dysfunction, and disrupted neurotransmitter signaling.

PD is a neurodegenerative disorder characterized by the progressive loss of dopaminergic neurons in the substantia nigra, leading to motor symptoms such as tremors, rigidity, and bradykinesia [16]. Alpha-synuclein aggregation: The aggregation of alpha-synuclein is a hallmark of PD pathology. Alpha-synuclein is a protein that usually plays a role in neurotransmitter release. Still, in PD, it misfolds and aggregates into toxic oligomers and fibrils that disrupt neuronal function and cause neurodegeneration [17]. Neuroinflammation: Chronic inflammation is a hallmark of PD pathology. Activated microglia and astrocytes produce inflammatory cytokines and chemokines, contributing to neurodegeneration [18]. In summary, the pathogenesis of PD involves multiple molecular and cellular mechanisms, including alpha-synuclein aggregation, mitochondrial dysfunction, neuroinflammation, impaired protein degradation, genetic factors, and disrupted neurotransmitter signaling. 

Stroke is a neurological disorder caused by interrupting blood flow to the brain, leading to brain damage and neurological deficits [19]. Here are some of the fundamental mechanisms involved. Ischemia: The primary cause of stroke is ischemia, which occurs when blood flow to the brain is interrupted due to a blockage or narrowing of a blood vessel. Ischemia decreases the oxygen and glucose supply to the brain, leading to energy failure and neuronal damage. Ischemic injury leads to the activation of microglia and astrocytes, releasing inflammatory cytokines and chemokines that exacerbate brain injury and contribute to secondary damage [20]. Ischemia, a reduced blood flow to an organ, including the brain, can cause a cascade of events leading to brain damage and cell death. Excitotoxicity occurs when the excessive release of glutamate leads to the overactivation of NMDA receptors, which can cause a buildup of calcium ions in the neurons, leading to cell damage and death. Oxidative stress occurs when the production of reactive oxygen species and free radicals overwhelms the brain’s antioxidant defense system, damaging cellular membranes, proteins, and DNA. Inflammation occurs when the immune system is activated, releasing cytokines, chemokines, and other inflammatory mediators, which can exacerbate brain damage and cell death. Finally, blood-brain barrier disruption can allow harmful substances, including immune cells and cytokines, to enter the brain, leading to further damage. Apoptosis: Ischemia leads to the activation of apoptotic pathways, leading to programmed cell death and contributing to brain injury. In summary, the pathogenesis of stroke involves multiple molecular and cellular mechanisms, including ischemia, excitotoxicity, oxidative stress, inflammation, BBB disruption, and apoptosis. 

PM_2.5_ is a significant component of air pollution and has been linked to various adverse health effects, including neurological diseases [21]. Inflammation: PM_2.5_ can activate the immune system, leading to the release of inflammatory cytokines and chemokines. Neurotoxicity: PM_2.5_ exposure has been linked to neurotoxicity, including neuronal damage, neuroinflammation, and cognitive impairment [22,23]. In summary, PM_2.5_ exposure can lead to various molecular and cellular mechanisms contributing to disease pathogenesis, including oxidative stress, inflammation, epigenetic modifications, mitochondrial dysfunction, autophagy impairment, and neurotoxicity. Understanding these mechanisms is critical for developing effective strategies to reduce the health effects of PM_2.5_ exposure. 

Research has found that chronic neuroinflammation is common in many neurodegenerative diseases (such as AD and PD) [3,24], stroke [25], and PM_2.5_ pollution [26]. It can contribute to the development and progression of these conditions by promoting neuronal damage and degeneration [6]. Overall, the pathophysiology of neuroinflammatory toxicity in these conditions is complex and not fully understood. Further research is necessary to fully comprehend the underlying mechanisms and identify potential targets for treatment and prevention.

## 2. Natural Products and Neuroprotection and Neuroinflammation

Many natural products have been found to have neuroprotective and anti-inflammatory effects, which may make them useful in treating neurodegenerative diseases and brain injuries [27,28,29]. For example, resveratrol found in grapes and berries, curcumin found in turmeric, and quercetin found in onions and apples have been shown to have neuroprotective effects. Quercetin is a naturally occurring flavonoid found in many fruits, vegetables, and nuts and is known to have various health benefits. Research has suggested that quercetin may help reduce the risk of chronic diseases, such as heart disease, diabetes, and certain types of cancer. Studies have shown that quercetin can activate AMPK, which can lead to several positive effects on cellular metabolism and health [30]. AMPK is a cellular energy sensor that is critical in regulating cellular metabolism and energy homeostasis. Here are some of the fundamental molecular and cellular mechanisms underlying the function of AMPK. AMPK activation: AMPK is activated by an increase in the AMP/ATP ratio, which occurs under conditions of energy stress, such as during exercise or nutrient deprivation. Upon activation, AMPK phosphorylates downstream targets involved in various cellular processes, including lipid and glucose metabolism, autophagy, and mitochondrial biogenesis. One of the critical functions of AMPK is to restore energy homeostasis by promoting catabolic pathways that generate ATP while inhibiting anabolic pathways that consume ATP. This is achieved by inhibiting anabolic pathways, such as protein and fatty acid synthesis, to conserve energy and promoting catabolic pathways, such as glycolysis and fatty acid oxidation, to generate ATP. AMPK also activates autophagy, a cellular process involved in degrading and recycling damaged or dysfunctional proteins and organelles. Autophagy is essential for maintaining cellular homeostasis and generating energy under nutrient deprivation or stress conditions. By activating autophagy, AMPK can generate power from the breakdown of cellular components and maintain cellular homeostasis.

**Figure 2:**
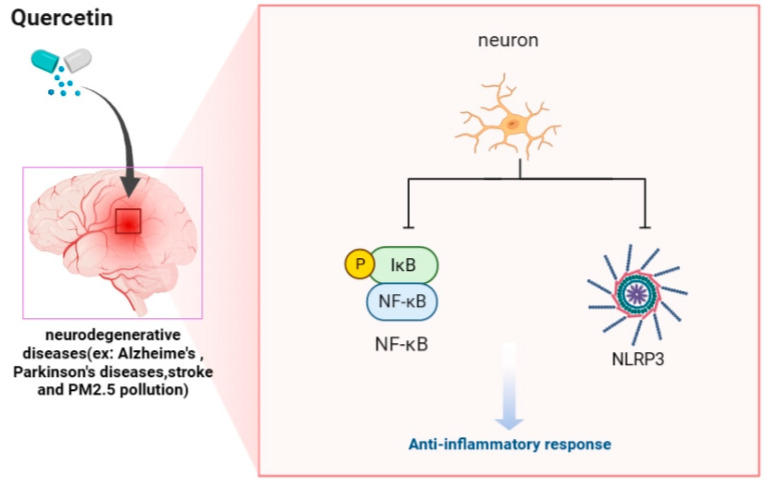
Quercetin is an anti-inflammatory agent for NF-kB and NLRP3 inflammasome in the brain of AD, PD, stroke, and PM_2.5_ pollution.

AMPK plays a critical role in regulating cellular metabolism and energy homeostasis by phosphorylating and altering the activity of downstream targets involved in lipid and glucose metabolism, autophagy, and mitochondrial biogenesis [31]. In addition, the activation of AMPK has emerged as a promising therapeutic target for various conditions associated with impaired energy homeostasis, and ongoing research continues to investigate the potential of AMPK activators as a treatment for these conditions. Understanding these mechanisms is essential for developing strategies to modulate AMPK activity for therapeutic purposes in metabolic disorders such as type 2 diabetes and neurologic diseases [32,33,34]. Activation of AMPK by quercetin has been shown to increase cellular energy production, reduce oxidative stress, and suppress the production of pro-inflammatory cytokines [30]. These effects may contribute to the anti-inflammatory and neuroprotective effects of quercetin. However, more research is needed to fully understand the mechanisms underlying the effects of quercetin on AMPK activation and neuroprotection and to determine the most effective dosing and administration strategies for quercetin. Quercetin is neuroprotective by inhibiting the activation of the NF-kB and NLRP3 inflammasome pathways (Figure 2). These findings suggest that quercetin may have potential therapeutic applications in treating neuroinflammatory conditions, such as AD, PD, stroke, and PM_2.5_ pollution.

Various signaling pathways, including the NF-kB pathway and the NLRP3 inflammasome, mediate inflammation in the brain. Both pathways have been implicated in the pathogenesis of neurodegenerative diseases and exposure to environmental pollutants. Quercetin may have potential therapeutic benefits for neurodegenerative diseases and exposure to environmental pollutants by targeting inflammation in the brain mediated by the NF-kB pathway and NLRP3 inflammasome. The image was created in BioRender.

### 2.1. Quercetin Is a Natural Anti-Inflammatory Agent That Alters Cellular Functions during Inflammation

Some studies have suggested that quercetin has neuroprotective effects due to its anti-inflammatory, antioxidant, and neuroprotective properties [35]. However, the research on quercetin’s effect on neuroinflammation toxicity and neuroprotection is still in its early stages, and more studies are needed to confirm these potential benefits. Quercetin has also been shown to have neuroprotective effects and to improve cognitive function in animal studies [36]. These findings suggest that quercetin may have potential benefits in reducing the damage caused by neuroinflammation and improving brain health. On the other hand, cellular functions are altered during inflammation, and quercetin has been shown to affect these changes. Studies have shown that quercetin can reduce the production of pro-inflammatory cytokines and have anti-inflammatory effects, suggesting that it may have potential benefits in reducing the cellular changes that occur during inflammation [37]. In addition, quercetin has been shown to have antioxidant properties, which may help to reduce oxidative stress and protect cells from damage during inflammation [38,39]. These findings suggest that quercetin may have potential benefits in reducing the cellular changes that occur during inflammation and improving the health of cells. AMPK pathways are a crucial regulator of cellular energy homeostasis and regulate various cellular processes, including metabolism, inflammation, cell growth and survival, and autophagy [40,41]. Inflammation is associated with a series of cellular changes, including oxidative stress, ER stress, and changes in cell signaling pathways. These changes can disrupt cellular homeostasis and activate AMPK, which plays a crucial role in regulating the cellular response to inflammation by modulating mitochondrial function, reducing oxidative stress, and inducing autophagy [42]. By doing so, quercetin and AMPK help to restore cellular homeostasis and prevent cellular damage during inflammation.

### 2.2. Neuroprotective Role of Quercetin in Neurodegenerative Diseases, Stroke, and PM_2.5_-Induced Neuroinflammatory Toxicity

Quercetin has been shown to have anti-inflammatory properties and has been studied in various neurodegenerative diseases such as AD, PD, stroke, and exposure to PM_2.5_ air pollution [35,37,43,44,45]. Some studies suggest that quercetin may have protective effects against neuroinflammation and potentially slow the progression of these diseases (Figure 2). However, more research is needed to fully understand the role of quercetin in neurodegenerative diseases and determine its efficacy as a treatment. Quercetin-activating AMPK has been shown to have beneficial effects on reducing neuroinflammation and improving neuronal function.

#### 2.2.1. The Effect and Molecular Mechanism of Quercetin in AD

The molecular mechanisms underlying the potential beneficial effects of quercetin in AD are complex and not fully understood. However, several studies have suggested that quercetin may exert its neuroprotective effects through various mechanisms. Quercetin has been shown to inhibit amyloid-beta (Aβ) aggregation and reduce the formation of Aβ plaques, which may help slow the progression of AD [46]. Quercetin has been shown to have anti-inflammatory effects by reducing the production of pro-inflammatory cytokines and other inflammatory molecules [47]. Regulation of gene expression: Quercetin has been shown to regulate the expression of genes involved in neuronal survival and synaptic plasticity, which may help protect against neuronal damage and improve cognitive function [48]. Modulation of signaling pathways: Quercetin has been shown to modulate several signaling pathways in the brain, including the PI3K/Akt and MAPK/ERK pathways [49]. These pathways play essential roles in neuronal survival, synaptic plasticity, and memory formation. Overall, the molecular mechanisms by which quercetin exerts its neuroprotective effects in AD are complex and multifactorial. While more research is needed to understand the mechanisms underlying its effects fully, quercetin may be a promising natural compound for preventing and treating AD.

Numerous papers discuss the potential therapeutic effects of quercetin in various diseases, particularly in AD and aging-related disorders, through mechanisms such as modulating inflammation, oxidative stress, and mitochondrial biogenesis. The following is a summary in Table 1. Zu et al. (2021) investigated the mechanism of quercetin in targeting therapeutic pathways for AD. They found that quercetin may exert neuroprotective and antidiabetic effects by modulating the activity of multiple enzymes and signaling pathways [50]. Cui et al. (2022) proposed that quercetin may have potential therapeutic benefits in aging-related diseases by activating the SIRT1 signaling pathway, which regulates cellular metabolism, oxidative stress, and aging [51]. Thiruvengadam et al. (2021) reviewed the potential of bioactive compounds, including quercetin, in modulating oxidative stress-mediated diseases by targeting the NRF2/ARE signaling pathway and epigenetic regulation [48]. Bayazid and Lim (2022) investigated the potential of quercetin in modulating the Nrf2/HO1 signaling pathway, which plays a crucial role in AD progression. They found that quercetin may protect against oxidative stress-mediated neuronal damage [43]. Wang et al. (2022) investigated the potential of quercitrin, a glycoside derivative of quercetin, in improving cognitive impairment in AD by inhibiting microglia-induced inflammation [47]. Ho et al. (2022) investigated the effect of quercetin on mitochondrial biogenesis and oxidative stress in neuronal cells. They found that quercetin may increase mitochondrial biogenesis and reduce free radicals in neuronal cells, potentially providing a protective effect against neuronal damage [52]. Chiu et al. (2023) investigated the potential neuroprotective effects of quercetin and apigenin in inhibiting Aβ aggregation and activating TRKB signaling in a cellular experiment. They found that quercetin and apigenin may protect against Aβ-induced neuronal damage [53]. These papers suggest that quercetin may have therapeutic potential in AD by modulating multiple signaling pathways involved in inflammation, oxidative stress, and neuronal damage.

#### 2.2.2. Mechanism of Quercetin Therapeutic Targets for PD

Quercetin has been shown to have potentially beneficial effects on PD by targeting various mechanisms involved in the pathogenesis of the disease [54]. Here are some of the results and molecular mechanisms of quercetin in PD. Anti-inflammatory effects: Quercetin has been shown to have anti-inflammatory effects by inhibiting the production of pro-inflammatory cytokines and other inflammatory molecules. Chronic inflammation in the brain is believed to contribute to the development and progression of PD, and reducing inflammation may help slow down the disease. Inhibition of alpha-synuclein aggregation: Alpha-synuclein is a protein that forms aggregates in the brains of individuals with PD. Quercetin has been shown to inhibit alpha-synuclein aggregation and reduce the formation of these aggregates, which may help slow down the progression of the disease [55]. Neuroprotection: Quercetin has been shown to have neuroprotective effects by protecting against neuronal damage and death in the brain. This may help slow the progression of PD and improve motor function. 

Several papers focus on the neuroprotective effects of quercetin in animal models of PD. The following is a summary in Table 2. Sharma et al. (2020) and Jain et al. (2022) investigated the effects of quercetin on rotenone-induced neuroinflammation and alterations in mice behavior [56,57]. Josiah et al. (2022) examined the effects of catechin and quercetin on dopamine metabolism and gene expression in male Wistar rats [58]. Ay et al. (2017) investigated the molecular mechanisms underlying the protective effects of quercetin against mitochondrial dysfunction and dopaminergic neurodegeneration in cell culture and MitoPark transgenic mouse models of PD [59]. Specifically, they highlight the ability of quercetin to mitigate the adverse effects of various toxins and stressors on neuronal health and function. The studies also investigate the underlying mechanisms of quercetin’s protective effects, including its ability to modulate dopamine metabolism and inflammation. Overall, these articles suggest that quercetin may potentially be a therapeutic agent for PD.

#### 2.2.3. Molecular Mechanisms Underlying Protective Role of Quercetin in Stroke

Quercetin has been investigated for its potential neuroprotective effects in stroke [60]. Here are some of the results and molecular mechanisms of quercetin in stroke. Inflammation plays a crucial role in the development and progression of a stroke, and reducing inflammation may help protect against neuronal damage. Quercetin has been shown to have anti-inflammatory effects by inhibiting the production of pro-inflammatory cytokines and other inflammatory molecules in stroke [61]. Inhibition of apoptosis: Quercetin has been shown to inhibit apoptosis, or programmed cell death, which can occur following stroke and contribute to neuronal damage. By preventing apoptosis, quercetin may help protect against neuronal damage and improve neurological outcomes after stroke. 

Some papers investigate the protective effect of quercetin against inflammatory neuronal injury and oxidative stress in different models of cerebral ischemia and hypoxic-ischemic brain injury. The following is a summary in Table 3. Wang et al. (2020) found that quercetin protects against cerebral ischemia/reperfusion injury and oxygen-glucose deprivation/reoxygenation neurotoxicity in rat hippocampal neurons by reducing oxidative stress and inflammation [62]. Park et al. (2020) showed that quercetin attenuates the decrease of thioredoxin expression following focal cerebral ischemia and glutamate-induced neuronal cell damage in rats. Thioredoxin is an antioxidant enzyme that protects cells against oxidative stress [63]. Le et al. (2020) demonstrated that quercetin alleviates neonatal hypoxic-ischemic brain injury in rats by inhibiting microglia-derived oxidative stress and TLR4-mediated inflammation. TLR4 is a receptor protein in the innate immune response [64]. Park et al. (2019) reported that quercetin alleviates the injury-induced decrease of protein phosphatase 2A subunit B in a rat model of cerebral ischemia and glutamate-exposed HT22 cells. Protein phosphatase 2A is a serine/threonine phosphatase that regulates multiple cellular processes [65]. Li et al. (2021) investigated the protective effect of quercetin on endothelial cells injured by hypoxia and reoxygenation. They found that quercetin reduces cell apoptosis and oxidative stress in human umbilical vein endothelial cells [66]. These studies suggest that quercetin has neuroprotective effects in different models of cerebral ischemia and hypoxic-ischemic brain injury by reducing inflammation and oxidative stress.

#### 2.2.4. Neuroprotective Effect and Molecular Mechanism of Quercetin on PM_2.5_

PM_2.5_ is a type of air pollutant linked to neurological disorders and cognitive impairment [67]. Here are some neuroprotective effects and molecular mechanisms of quercetin on PM_2.5_. PM_2.5_ exposure can lead to inflammation in the brain, and reducing inflammation may help protect against neuronal damage. Quercetin has been shown to have anti-inflammatory effects by inhibiting the production of pro-inflammatory cytokines and other inflammatory molecules in PM_2.5_ [68]. Regulation of gene expression: Quercetin has been shown to regulate the expression of genes involved in inflammation, oxidative stress, and other processes that may be affected by PM_2.5_ exposure. This may help reduce the harmful effects of PM_2.5_ on the brain. 

Various papers investigate the protective effects of quercetin against inflammation and oxidative damage induced by exposure to PM_2.5_ during gestation in mice and human bronchial epithelial cells. The following is a summary in Table 4. Zhang et al. (2018) found that maternal exposure to PM_2.5_ during gestation induced neurodevelopmental toxicity in rat pups, which was attenuated by treatment with quercetin. The protective effects of these antioxidants were attributed to their ability to reduce inflammation and oxidative stress [69]. Jin et al. (2016) demonstrated that quercetin attenuated PM_2.5_-induced oxidative damage in human bronchial epithelial cells by reducing reactive oxygen species production and increasing antioxidant enzyme activity [70]. Liu et al. (2017) investigated the effects of quercetin on maternal immunity, inflammation, and oxidative stress in mice exposed to fine particulate matter during gestation. They found that quercetin reduced inflammation and oxidative stress in the maternal lungs and placenta and improved the immune function of the offspring [68]. Liu et al. (2019) reported sex-dependent effects of PM_2.5_ maternal exposure and quercetin intervention on offspring’s short-chain fatty acids in rats. They found that quercetin intervention increased the levels of short-chain fatty acids in male but not female offspring, indicating that the protective effects of quercetin may be sex-dependent [71]. Overall, these studies suggest that quercetin protects against inflammation and oxidative damage induced by PM_2.5_ exposure. 

## 3. Quercetin Is an Anti-Inflammatory Agent via AMPK and Is Neuroprotective for NF-kB and NLRP3 Inflammasome in Neuroinflammatory Toxicity

**Figure 3:**
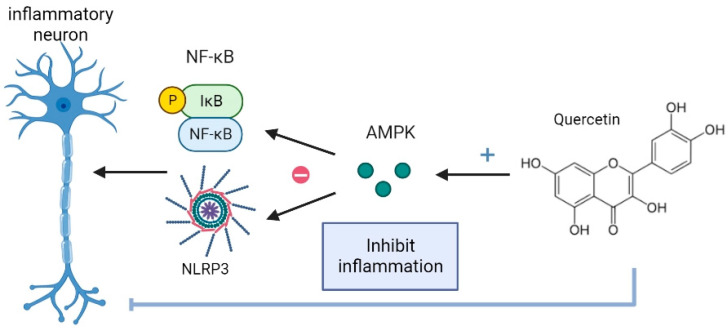
Quercetin is an anti-inflammatory agent via AMPK and is neuroprotective for NF-kB and NLRP3 inflammasome in neuroinflammatory toxicity. AMPK activation by quercetin can inhibit the production of pro-inflammatory cytokines and chemokines, such as TNF-α, IL-1β, and IL-6, by inhibiting NF-κB and NLRP3 inflammasome signaling pathways. Quercetin’s anti-inflammatory and neuroprotective effects make it a promising candidate for treating neuroinflammatory disorders, including those associated with exposure to environmental toxins. The image was created in BioRender.

Quercetin has been shown to have anti-inflammatory and neuroprotective effects through its modulation of AMPK and its impact on the NF-kB and NLRP3 inflammasome pathways (Figure 3). NF-kB is a transcription factor that regulates gene expression in inflammation, cell survival, and other biological processes. The NF-kB signaling pathway is involved in regulating pro-inflammatory cytokines and oxidative stress. Quercetin has been shown to suppress NF-kB activation, which may contribute to its anti-inflammatory effects [72,73]. Quercetin has been shown to suppress the activation of the NLRP3 inflammasome, which may contribute to its neuroprotective effects [74]. The NLRP3 inflammasome is a cytosolic complex composed of several proteins, including NLRP3, ASC, and caspase-1, that play a crucial role in regulating the immune response to various forms of cellular stress and danger signals [75,76]. Activation of the NLRP3 inflammasome leads to the activation of caspase-1, which cleaves pro-inflammatory cytokines, such as interleukin-1 beta (IL-1β) and interleukin-18 (IL-18), and triggers their release into the extracellular space. The NLRP3 inflammasome is a complex of proteins involved in regulating inflammation and cell death. Overall, these findings suggest that quercetin has the potential to be an effective neuroprotective agent by modulating critical cellular pathways involved in the regulation of NF-kB and NLRP3 inflammasome in the context of neuroinflammatory toxicity [51,77]. 

### 3.1. Quercetin via AMPK Suppresses NF-κB and NLRP3 Inflammasome Activation in AD and PD

There is growing evidence to suggest that quercetin has anti-inflammatory and antioxidant properties, which may be beneficial for preventing and treating AD. In AD, NF-kB is thought to be activated in microglia and astrocytes, leading to the production of pro-inflammatory cytokines and chemokines, which contribute to the progression of the disease. Quercetin has been shown to suppress the activation of NF-kB in these cells, thereby reducing inflammation in the brain [78]. In addition, the NLRP3 inflammasome is another important inflammatory pathway thought to be activated in AD [79]. The inflammasome is a protein complex that triggers the release of pro-inflammatory cytokines, including interleukin-1β (IL-1β), in response to various stimuli, including Aβ peptides, a hallmark of AD. Quercetin, with anti-inflammatory properties, has been suggested to have therapeutic potential for PD [78]. Activation of NF-kB has been shown to promote neuroinflammation, which contributes to the progression of PD. Quercetin has been shown to suppress the activation of NF-kB in the brain, thereby reducing inflammation in PD [80]. Studies have shown that quercetin can activate AMPK, which can suppress the activation of two critical inflammatory pathways, NF-kB and NLRP3 inflammasome, in the CNS [77,81]. Overall, these findings suggest that quercetin may have therapeutic potential for treating AD and PD by suppressing the activation of NF-kB and NLRP3 inflammasome, two key inflammatory pathways that contribute to the pathogenesis of the disease [74,82,83]. 

### 3.2. Quercetin Inhibits NF-κB and NLRP3 Inflammasome in Stroke and PM_2.5_ by AMPK Signaling Pathway

Quercetin has been shown to inhibit the activation of NF-kB and NLRP3 inflammasome by activating the AMPK signaling pathway, which may have potential therapeutic implications for stroke [60]. Quercetin has been shown to suppress the activation of NF-kB in the brain, thereby reducing inflammation in stroke [60]. NLRP3 inflammasome is another important inflammatory pathway that is activated in stroke. Activation of AMPK by quercetin leads to the suppression of NF-kB and NLRP3 inflammasome activation, which in turn reduces inflammation in stroke. PM_2.5_-induced inflammation is associated with the activation of NF-kB and NLRP3 inflammasome, which produces pro-inflammatory cytokines and develops oxidative stress [22,84,85,86]. Quercetin has been shown to inhibit PM_2.5_-induced inflammation by inhibiting the activation of NF-kB and NLRP3 inflammasome through the AMPK signaling pathway [87]. Overall, the anti-inflammatory effects of quercetin via the AMPK signaling pathway may have potential therapeutic implications for stroke and PM_2.5_.

## 4. Conclusions

Neuroinflammation is a complex process that involves the activation of various immune cells, such as microglia and astrocytes, and the production of pro-inflammatory cytokines and chemokines in response to different insults, including neurodegenerative diseases, strokes, or exposure to environmental toxins. Neuroprotection is the strategy that aims to prevent or reduce neuronal damage and promote neuronal survival in various neurological disorders. Neuroprotection can involve targeting different pathways, such as anti-inflammatory and antioxidant pathways, to avoid or minimize neuroinflammation and oxidative stress. Quercetin has potent anti-inflammatory and antioxidant properties, making it a potential candidate for neuroprotection and neuroinflammation. In neurodegenerative diseases, such as AD, quercetin has been shown to have neuroprotective effects by reducing beta-amyloid aggregation and neuroinflammation. In PD, quercetin has been shown to have neuroprotective effects by reducing neuroinflammation and oxidative stress in the substantia nigra. Therefore, this brain region is primarily affected by PD. In stroke, quercetin has been shown to have neuroprotective effects by reducing brain damage and improving neurological outcomes. In PM_2.5_-induced neuroinflammatory toxicity, quercetin has been shown to have neuroprotective effects by reducing neuroinflammation, oxidative stress, and apoptosis. Quercetin has shown promising potential as a natural product for neuroprotection and neuroinflammation in various neurological disorders.

Quercetin is a natural flavonoid that has been shown to have anti-inflammatory and neuroprotective effects, partly through its ability to modulate the activity of specific signaling pathways. One of the mechanisms by which quercetin exerts its anti-inflammatory effects is activating AMPK, an energy sensor in cells that helps balance energy production and utilization. Activation of AMPK by quercetin has been shown to inhibit the activation of pro-inflammatory signaling pathways, including NF-κB, a key regulator of inflammation. In addition, quercetin has also been shown to exert a neuroprotective effect by suppressing the activation of the NLRP3 inflammasome, which is involved in the development of neuroinflammatory toxicity. By inhibiting the activation of this complex, quercetin can help to reduce the release of pro-inflammatory cytokines and limit the damaging effects of inflammation on neuronal cells. Overall, these findings suggest that quercetin may have the potential as a therapeutic agent for the treatment of neuroinflammatory conditions and other chronic inflammatory diseases.

## Figures and Tables

**Table 1:** Quercetin has neuroprotective and anti-inflammatory effects, reducing the production of pro-inflammatory cytokines in AD.

Biological Model	Pathways	Targets/Mechanisms	References
Quercetin can simultaneously interfere with AD progression (in vitro)	MAPK signaling	Regulate AKT1, JUN, MAPK, TNF, VEGFA, and EGFR	[50]
Quercetin (100 mg/kg) exerts neuroprotective effects against chronic aging-related diseases in AD mice model (in vivo)	Oxidative stress, Inflammatory, Mitochondrial damage and Autophagy.	Inhibited SIRT1/Keap1/Nrf2/HO-1 pathway, PI3K/Akt/GSK-3β, SIRT1/NF-Κb pathway, SIRT1/PGC1α/eIF2α/ATF4/CHOP pathway, and SIRT1/FoxO pathway	[51]
Quercetin (100 mg/kg) exhibited a synergistic effect with sitagliptin and improved cognitive memory in the rat brain (in vivo)	Decreasing the Aβ_1-42_ levels, enhancing the antioxidant activity	Increasing the expression of the NRF2/ARE pathway	[48]
Aβ and hippocampal tau phosphorylation were reduced during quercetin treatment (in vivo)	Protect neuronal cell death	Modulating Nrf2/HO-1 pathways	[43]
Quercitin (50 or 100 mg/kg) improved 5XFAD mice’s cognitive impairment (in vivo)	Anti-inflammatory response	Inhibited IL-1α, IL-6, IL-17A, G-CSF, IL-4, CXCL-1, Eotaxin, G-CSF, MIP-1α and MIP-1β	[47]
Quercetin (2.5, 5.0, 7.5, and 10.0 μM) increases mitochondrial biogenesis in hydrogen peroxide (H2O2)-induced oxidative stress neuronal SH-SY5Y cells (in vitro)	ROS production and mitochondrial biogenesis	Elevating the activity of the SIRT1-PGC-1α-TFAM pathway	[52]
Quercetin (100 μM) improved neurite outgrowth and reduced caspase-1/AChE activities in Aβ-GFP SH-SY5Y cells (in vitro)	Regulating neuronal survival and oxidative stress	Activating TRKB, enhancing NRF2, and reducing ROS	[53]

**Table 2:** Quercetin has neuroprotective and anti-inflammatory effects, reducing the production of pro-inflammatory cytokines in PD.

Biological Model	Pathways	Targets/Mechanisms	References
Quercetin (25 mg/kg) had a neuroprotective effect against rotenone- and iron supplement-induced PD in experimental rats (in vivo)	Anti-inflammatory, antioxidant, and neuroprotective effect	Improve biochemical (LPO, nitrite, GSH, mitochondrial complexes I and IV), neuroinflammatory (TNF-α, IL-1β, and IL-6), and neurotransmitter (dopamine, norepinephrine, serotonin, GABA, glutamate)	[56]
Quercetin (30 mg/kg) has a neuroprotective effect against rotenone-induced neuroinflammation and alterations in PD-like symptoms and mice behavior (in vivo)	Anti-neuroinflammation, improved memory, and cognitive function	Regulate the release of inflammatory markers in blood serum, astrocytes activation in substantia nigra and hippocampus, and subsequently decreased density of dopaminergic fibers in the striatum	[57]
Quercetin (5–20 mg/kg) has neuroprotective effects in an experimental model of Parkinsonism in male Wistar rats (in vivo)	Modulate dopamine metabolism and decrease neuroinflammation through the downregulation of pro-inflammatory cytokines and genes involved in inflammation and cell death pathways	Attenuating effect on NF-κB mediated inflammation (IL-1β, TNF-α, NF-κB, and IκKB) and the pro-apoptotic gene (p53)	[58]
Quercetin (10 and 30 μM; 25 mg/kg)has protective effects against mitochondrial dysfunction and progressive dopaminergic neurodegeneration in MN9D dopaminergic neuronal cells and MitoPark transgenic mouse models of PD (in vivo)	Mediate neuroprotective signaling and mitochondrial bioenergetics capacity	Induced the activation of two major cell survival kinases, protein kinase D1 (PKD1) and Akt; enhanced cAMP response-element binding protein phosphorylation and expression of the cAMP response-element binding protein target gene brain-derived neurotrophic factor	[59]

**Table 3:** Quercetin has been shown to have neuroprotective and anti-inflammatory effects in stroke.

Biological Model	Effects	Targets/Mechanisms	References
Quercetin (25 mg/kg) protects against cerebral ischemia injury and oxygen-glucose deprivation neurotoxicity in SD rats and neuron/glia cultures (in vivo and in vitro)	Neuroprotective, anti-oxidative, anti-inflammatory, and anti-apoptotic effects	Biochemical studies revealed a reduction of ERK and Akt phosphorylation, TNF-α and IL-1β mRNA expression, along with apoptotic caspase 3 activity	[62]
Quercetin (10 mg/kg) has the potential as a neuroprotective agent and protects against oxidative stress and neuronal damage in cerebral ischemia SD rats and primary cultures of neurons (in vivo and in vitro)	Neuroprotective against oxidative stress and neuronal damage.	Regulate thioredoxin expression and maintain interaction between ASK1 and thioredoxin	[63]
Quercetin (50 mg/kg) inhibited oxygen-glucose deprivation-induced expression of inflammatory factors in BV2 cells and suppressed cerebral infarct volume in oxygen-glucose deprivation mice (in vivo and in vitro)	Anti-inflammatory and anti-oxidative effects	Inhibition of TLR4-mediated inflammatory responses and oxidative stress in activated microglia	[64]
Quercetin (10 mg/kg) has the potential as a neuroprotective agent by alleviates cerebral ischemic animal models and glutamate-exposed HT22 cells (in vivo and in vitro)	Neuroprotective function in ischemic brain injury	Increase the expression of PP2A-B and protect against neuronal injury and cell death	[65]
Protective Effect of Quercetin (1 μM) on human brain microvascular endothelial cells injured by hypoxia damage (in vitro)	Inhibition of endoplasmic reticulum stress and antioxidation	Promote the Keap1/Nrf2 signaling pathway, and reduce ATF6/GRP78 protein expression	[66]

**Table 4:** Quercetin has been shown to have neuroprotective and anti-inflammatory effects in exposure to PM_2.5_.

Biological Model	Effects	Targets/Mechanisms	References
Quercetin protects against PM_2.5_-induced neurodevelopmental toxicity in animal models (in vivo)	Oxidative stress, inflammatory response, and modulation of the CREB/BDNF signaling pathway	Improve BDNF, TrkB, p-CREB/CREB, p-Akt/Akt, p-ERK1/2/ERK1/2 expression	[69]
Quercetin has an inhibitory effect on PM_2.5_-induced respiratory oxidative damage and inflammation in human bronchial epithelial cells (in vitro)	Antioxidant and anti-inflammation properties	Regulates NADPH oxidase, inflammation cytokines, SIRT1, p53R2, NDUFS2, and UQCRI1 levels	[70]
Quercetin (100 mg/kg) has protective effects against the adverse effects of PM_2.5_ exposure in Pregnant mice (in vivo)	Anti-inflammatory and antioxidant properties	Inhibit biomarkers of systemic inflammation injuries (IL-2, IL-6, IL-8, and TNF-α) and oxidative stress indicators (CAT, GSH, and HO-1)	[68]
Quercetin (100 mg/kg) intervention during gestation protects against the adverse effects of PM_2.5_ exposure (in vivo)	Anti-inflammatory and antioxidant properties	Quercetin administration during gestation has been shown to have the potential to offset the effects of maternal PM_2.5_ exposure on short-chain fatty acids in offspring	[71]

## Data Availability

Not applicable.
